# The Bistable Behaviour of *Pseudomonas putida* KT2440 during PHA Depolymerization under Carbon Limitation

**DOI:** 10.3390/bioengineering4020058

**Published:** 2017-06-19

**Authors:** Stephanie Karmann, Sven Panke, Manfred Zinn

**Affiliations:** 1 Institute of Life Technologies, University of Applied Sciences and Arts Western Switzerland (HES-SO Valais), Route du Rawyl 47, 1950 Sion, Switzerland; stephanie.karmann@bsse.ethz.ch; 2 Department of Biosystems Science and Engineering, ETH Zurich (ETHZ), Mattenstrasse 26, 4058 Basel, Switzerland; sven.panke@bsse.ethz.ch

**Keywords:** *Pseudomonas putida*, flow cytometry, polyhydroxyalkanoate, PHA mobilization, PHA depolymerization, starvation, carbon limitation, BODIPY 493/503

## Abstract

Poly(hydroxyalkanoates) (PHAs) are bacterial polyesters offering a biodegradable alternative to petrochemical plastics. The intracellular formation and degradation of PHAs is a dynamic process that strongly depends on the availability of carbon and other nutrients. Carbon excess and nitrogen limitation are considered to favor PHA accumulation, whereas carbon limitation triggers PHA depolymerization when all other essential nutrients are present in excess. We studied the population dynamics of *Pseudomonas putida* KT2440 at the single cell level during different physiological conditions, favoring first PHA polymerization during growth on octanoic acid, and then PHA depolymerization during carbon limitation. PHAs accumulate intracellularly in granules, and were proposed to separate preferentially together with nucleic acids, leading to two daughter cells containing approximately equal amounts of PHA. However, we could show that such *P. putida* KT2440 cells show bistable behavior when exposed to carbon limitation, and separate into two subpopulations: one with high and one with low PHA. This suggests an asymmetric PHA distribution during cell division under carbon limitation, which has a significant influence on our understanding of PHA mobilization.

## 1. Introduction

In nature, bacteria rarely live under constant nutrient conditions. It is a clear advantage for single cell organisms to accumulate and store carbon if other nutrients are growth-limiting. This stored carbon can later be mobilized as an energy source for growth when all other essential nutrients are present again. Many bacteria can store carbon intracellularly in the form of poly(hydroxyalkanoate) (PHA) granules [1,2]. This polyester receives much attention as a biodegradable alternative to petrochemical plastics [3,4]. PHAs are applied as biodegradable packaging materials in the food industry [5], or in the biomedical field as biocompatible medical implants, biodegradable sutures, skin substitutes, and much more [6].

*Pseudomonas putida* contains two independent enzymes that are responsible for PHA polymerization, PhaC1 and PhaC2, and one responsible for depolymerization, the depolymerase PhaZ. The polymerizing and depolymerizing enzymes are directly associated with the PHA granule and form the so-called carbonosomes [7]. The PHA polymerization and depolymerization processes are closely linked to enable the immediate adaptation to changes in the chemical composition of the cells’ environment [8,9].

The intracellular polymer content of a bacterial culture can be quantified with different techniques, such as gas chromatography (GC) [10,11], fluorescence microscopy [12,13,14], or flow cytometry (FCM) [15,16]. Acomprehensive review about qualitative and quantitative methods for PHA analytics has been published recently [17]. Most quantification methods allow for determining an average amount of PHA per cell dry weight or similar. FCM is the only method that enables a quantification of intracellular PHA at the level of subpopulations and single cells. Single cell analysis is of particular interest for the characterization of biological samples such as PHA producing bacteria, since even genetically identical cells show considerable diversity in their actual composition (e.g., RNA and protein level), and effectively different physiological states can co-exist (bistability) [18]. Such bistability has been reported for *Bacillus subtilis* and its expression of the sporulation gene *spoIIA*. The FCM measurements of green fluorescent protein expressed under a *spoIIA* promotor revealed two distinct populations with a high and a low level of SpoIIA when entering the stationary phase. Effectively, only the high level SpoII population entered sporulation [19].

There are several publications about intracellular PHA quantification by FCM [15,16,20,21]. Interestingly, none of them compared the distribution pattern of PHA granules among the cells under different growth conditions. Typically, the PHA granule-associated phasin proteins are involved in the structural aspects of the PHA granule [22,23,24]. In particular, it has been suggested for *P. putida* that the PhaF phasin plays a crucial role for the distribution of PHA granules into the two new daughter cells upon cell division. PhaF has a DNA and PHA binding domain, and was suggested to ensure that the PHA is separated together with the chromosomes in a homogeneous way [25]. A similar behavior was reported for poly(hydroxybutyrate) (PHB) producing *Ralstonia eutropha* wild type cells, where the granules were located in the vicinity of the nucleoid region and separated in a homogeneous way to the daughter cells [26].

Here, we show by a FCM-based single cell analysis of *P. putida* KT2440 cultures that the onset of carbon limitation leads in fact to the appearance of two subpopulations of bacteria: one which seems to retain the PHA granules, and one that seems to receive none or at least substantially less than the first one. This suggests that, at least at this stage, PHA distribution is in fact highly asymmetrical, which casts doubt on the generality of the previously postulated equal distribution mechanism.

## 2. Materials and Methods

### 2.1. Bacterial Strains, Growth Conditions, and Media

All experiments were performed with *P. putida* KT2440 (ATCC 47054) stored in 1.5 mL 16% glycerol stocks at −80 °C. The strain was grown on LB plates [27] to inoculate precultures as described previously [15]. The modified mineral medium E2 with a pH of 7 [28] and a reduced ammonium content [15] was used for liquid cultivation. Per L of dH_2_O were added: 7.5 g K_2_HPO_4_, 3.7 g KH_2_PO_4_, and 1.49 g NaNH_4_HPO_4_·4H_2_O. Trace elements and MgSO_4_·7H_2_O were added as published [28]. The carbon sources were supplied depending on the application of the medium: the preculture medium contained 1.89 g·L^−1^ of citrate and 0.144 g·L^−1^ of octanoic acid (C8), whereas the initial medium for fed-batch fermentations contained 0.189 g·L^−1^ of citrate.

### 2.2. Bioreactor Settings and Growth Conditions for Fed-Batch Fermentations

The fed-batch fermentation was carried out in a 3.6 L bioreactor (Labfors 5, Infors AG, Bottmingen, Switzerland) with an initial working volume of 2.5 L. Online probes were continuously recording pH and pO_2_ (Hamilton Bonaduz AG, Bonaduz, Switzerland). The pH was maintained at 7.0 ± 0.05 by the automated addition of 3 M KOH or 3 M H_3_PO_4_. The culture was agitated with two six-blade Rushton turbines (diameter of 54 mm) and aerated with normal air at 1 L·L^−1^·min^−1^. To maintain a pO_2_ above 45%, the agitation was programmed in a cascade mode allowing a stirrer speed between 600 and 1000 rpm. An aliquot of 1 mL PPG2000 antifoam was added to the initial medium to avoid foam formation. The temperature was kept constant at 30 °C.

To inoculate the bioreactor, 150 mL of a preculture with an OD_600_ of 2.0 were harvested, centrifuged (4400 *g*, 4 °C, 30 min), and resuspended in 20 mL 0.1 M K_2_HPO_4_ buffer of pH 7. The initial OD_600_ in the bioreactor was 0.1.

The fed-batch fermentation consisted of three different phases: first, an exponential feeding, followed by a linear feeding, and finally no feeding at all in order to reach carbon limitation. The exponential feeding of an aqueous solution containing 0.75 M C8 and equimolar KOH was controlled by a liquid mass flow controller (mini Cori Flow M12, Bronkhorst Cori-Tech B.V., Ruurlo, The Netherlands). The exponential feed rate was programmed with the system software Iris (Infors AG), leading to a specific growth rate of 0.3 h^−1^, which corresponded to 50% of the maximum specific growth rate (µ_max_) of *P. putida* KT2440 on C8, as determined in previous experiments with *P. putida* KT2440 [15]. After 12.75 h, nitrogen limitation was reached and the feed rate was kept constant for 3.75 h at 1.48 g·h^−1^ of C8. The feeding resulted in a carbon to nitrogen (C/N) ratio of 26 g·g^‒1^. The third phase, without carbon feeding, started after 16.3 h of fermentation. At the same time, an ammonium pulse was applied in the form of Na(NH_4_)HPO_4_·4H_2_O (20.9 g in 100 mL dH_2_O) that was aseptically added through a sterile 0.22 µm filter (Sarstedt, Nümbrecht, Germany). This pulse reduced the C/N ratio to 4 g·g^−1^.

Frequent samples were taken throughout the bioprocess. The optical density and FCM measurements were done immediately with fresh culture. The supernatant samples to determine the ammonium nitrogen (NH_4_^+^-N) or C8 content were stored at −20 °C. The cell pellets for cellular PHA quantification by GC were stored at −80 °C.

The fed-batch was run twice with the same feeding strategy, resulting in two identical data sets. For clarity, only one is shown.

### 2.3 Analytics

#### 2.3.1. Biomass, NH_4_^+^-N Quantification

To follow the growth of the culture, the cellular dry weight (CDW) was measured by weighing a triplicate of 2 mL dried culture broth as described previously [29], and the optical density was measured at 600 nm (OD_600_) [15]. The NH_4_^+^-N in the culture supernatant was determined with a test kit (Ammonium-Test Spectroquant, Merck, Darmstadt, Germany) [15].

#### 2.3.2. Quantification of Octanoic Acid (C8)

The C8 content in the supernatant was determined by high pressure liquid chromatography (HPLC). A volume of 10 µL of undiluted supernatant (centrifuged 30,670 *g*, at 4 °C for 5 min) was injected into a chromatograph (1200 Series, Agilent, Santa Clara, CA, USA) equipped with a Zorbax Eclipse XDB-C18, 150 × 4.6 mm^2^, 5 µm (Agilent Nr 993967-902) and a 12.5 × 4.6 mm^2^, 5 µm guard column (Agilent Nr 820950-925). A gradient elution with 0.1% *v*/*v* formic acid in acetonitrile (eluent A) and 0.1% *v*/*v* formic acid in milliQ water (eluent B) was applied with a linear gradient from 30 to 100% of eluent A within the first 10 min followed by 15 min of elution with 100% of eluent A. The temperature of the column was kept at 25 °C. Finally, the detection was performed with a 210/4 nm UV detector.

#### 2.3.3. Quantification of Volumetric Total Cell Count (vTCC) and PHA by FCM

The *P. putida* KT2440 culture was sampled in duplicates, diluted, and then double-stained with BODIPY 493/503 and SYTO 62 according to a previously published protocol [15]. FCM was carried out with a BD Accuri C6 flow cytometer (BD Bioscience, Erembodegem, Belgium). The results are shown as mean values from the two samples, which had a maximal difference of 10%.

#### 2.3.4. PHA Quantification by GC

The samples of the culture broth with a volume between 10 and 300 mL were centrifuged (3900 *g*, 4 °C, 10 min), and washed once with 0.9% aqueous NaCl. The cell pellet was stored at −80 °C for at least 24 h, then freeze-dried and later methanolyzed as described earlier [11,15].

## 3. Results and Discussion

### 3.1. Fed-Batch with Feeding Phase and Subsequent Carbon Limitation Inducing PHA Polymerization and Depolymerization

**Figure 1:**
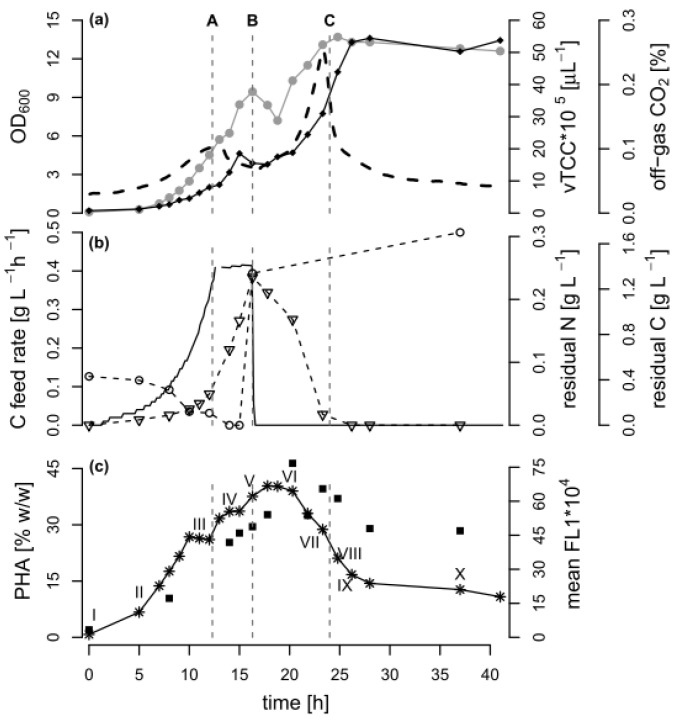
Bioprocess data of the fed-batch fermentation. A fed-batch cultivation of *Pseudomonas putida* KT2440 was carried out with an exponential feed of octanoic acid (C8), followed by a poly(hydroxyalkanoate) (PHA) depolymerization phase under carbon limitation. The dashed lines indicate A: the time point 12.3 h when the feed changed from exponential to linear mode, B: the time point 16.3 h when the carbon feeding was stopped and an ammonium pulse was added, and C: the time point 24 h when the carbon source was depleted. (**a**) Optical density (•, OD_600_), volumetric total cell count (♦ vTCC), CO_2_ content in the off-gas (--); (**b**) Carbon (C) feed rate (-), carbon and nitrogen content in the culture supernatant (∇ residual C, ◦ residual N); (**c**) PHA quantity as determined by gas chromatography (GC) analysis (∎) and by measuring the mean green fluorescence (✴ mean FL1) of BODIPY 493/503 stained cells with flow cytometry (FCM). The Roman numbers (I–X) in proximity of the mean FL1 values mark the FCM samples depicted in Figure 3 in Section 3.3.

We performed a fed-batch process with *P. putida* KT2440 that consisted of an initial PHA accumulation phase (carbon excess and nitrogen limitation, 0–16.3 h), followed by a depolymerization phase (nitrogen excess and later carbon limitation, ~20–40 h). During this fermentation, the growth and the PHA-based population structure were analyzed by FCM (Figure 1).

In general, after 12.3 h of exponential C8 feeding, an OD_600_ of 5.7 was reached. Subsequently, a linear carbon feed was applied over 4 h to increase PHA accumulation under nitrogen limitation. During this time, a total amount of 2.67 g·L^−1^ carbon was supplied to the culture, leading to a reduction of the nitrogen concentration in the culture supernatant below the detection limit after 13 h. A pulse of ammonium was added after 16.3 h, resulting in a concentration in the culture broth of 0.3 g NH_4_^+^-N L^−1^. With this nitrogen excess, the remaining carbon source was completely consumed after 24 h. The cultivation continued for 17 h under the carbon-limited condition.

### 3.2. Comparison of PHA Quantification by FCM and GC during PHA Polymerization and Depolymerization

The cellular PHA content was followed by FCM and GC throughout all of the fed-batch phases. In FCM-based single cell analytics, the dye BODIPY 493/503 can be used to stain the PHA granules so that the green fluorescence (mean FL1) value per cell can be taken as a measure of the accumulated PHA [15]. As expected, the mean green fluorescence increased as long as C8 but no nitrogen was present in the medium, suggesting that the PHA amount per cell increased. When nitrogen was available again, and later when the carbon source was depleted, the PHA-derived fluorescence decreased, and reached a plateau at around 30% of the maximum value after 27 h of cultivation. The GC analysis of selected biomass samples confirmed that the PHA content was maximal between 17.5 and 20 h of cultivation (46% *w*/*w*). The accumulated PHA was a co-polymer of 3-hydroxyoctanoate and 10–11 mol % 3-hydroxyhexanoate.

**Figure 2:**
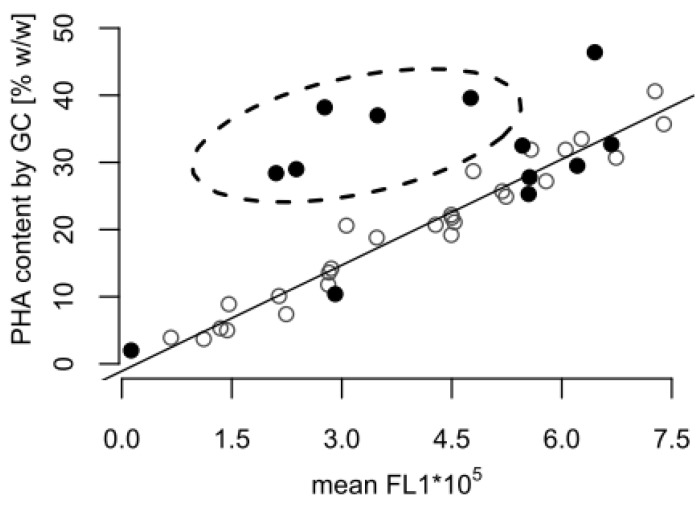
Correlation between poly(hydroxyalkanaote) (PHA) contents determined by gas chromatography (GC) and flow cytometry (FCM). The plot contains PHA content data measured by GC and FCM from the fed-batch shown in Figure 1 (•), and from previously published fed-batches with *P. putida* KT2440 grown on octanoic acid without a carbon-limited phase (◦) [15]. The FCM data are given as mean green fluorescence derived from BODIPY 493/503 stained intracellular PHA (mean FL1). The dashed ellipse comprises all samples taken during the carbon-limited phase of the fed-batch fermentation (Figure 1), which significantly differ from the samples taken during growth with carbon in excess.

Towards the end of the process, the GC analysis suggested that the cellular PHA content remained at a high level (almost 30% *w*/*w*), whereas a content of approximately 10% *w*/*w* would be expected based on the fluorescence data alone (Figure 1). Figure 2 shows that the correlation between the FCM and GC measurements was consistent with the linear correlation equation identified earlier [15], confirming that the FCM method is a robust method with hardly any inter-experimental variations. However, the samples taken in the experiments presented here during the depolymerization phase separated clearly from this line (Figure 2). The results indicate that either the FCM method underestimates the actual PHA content during the depolymerization phase, or the GC-based method overestimates it.

An explanation for the mismatch between the FCM and GC-based PHA amounts might be presented by the different mechanisms for PHA determination. A GC analysis will also include PHA degradation products, such as 3-hydroxyoctanoic acid monomers accumulated intracellularly or attached to the cell membrane, as well as PHA granules released from lysed cells. In order to avoid that GC measurements could be biased in such a way, the washing of the cell pellet prior to freeze-drying was an important step of the sample’s preparation protocol. However, the efficiency of the removal of water insoluble particles was not quantified, and it cannot be excluded as a source of error. In contrast, the double-staining-based FCM method applied in this study only considers PHA in intact cells. Released PHA granules and cell debris are ignored in the FCM measurement, as they do not contain any DNA, and consequently are not stained in the double staining procedure with the red fluorescent SYTO 62 [15].

### 3.3. Population Dynamics of P. putida KT2440 during PHA Polymerization and Degradation Determined by FCM

**Figure 3:**
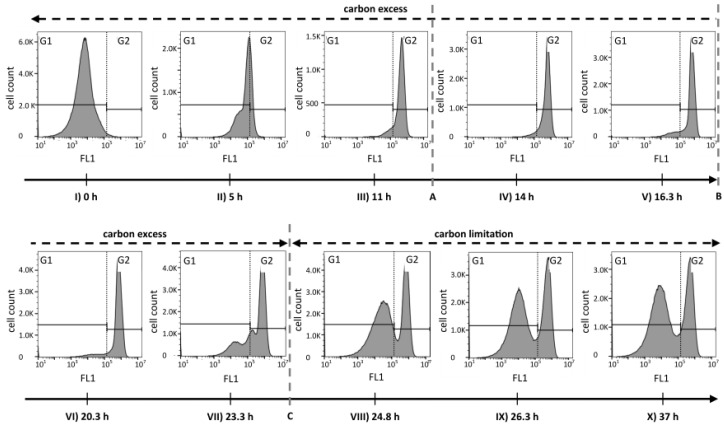
Histograms of fluorescence data (FL1) representing the PHA distribution among *P. putida* KT2440 cells during a fed-batch with a phase of carbon excess and a phase of carbon limitation. The green fluorescence of BODIPY 493/503 stained cells was recorded by flow cytometry during the bioprocess that is depicted in Figure 1. The dashed lines A, B, and C correspond to the time points (**A**) 12.3 h feed changed from exponential to linear mode; (**B**) 16.3 h the carbon feeding was stopped and an ammonium pulse was added; and (**C**) 24 h the carbon source was depleted. The gates G1 and G2 (black step lines and dotted vertical line) separate low and high FL1 values, respectively, and were defined based on the histogram from PHA-free cells at the time of inoculation, to visualize the evolution of the FL1 signal.

The analysis of FCM fluorescence histograms from selected time points acquired during different phases of the cultivation suggested that the PHA content changed quite drastically over time (Figure 3). During growth under carbon excess (data points I to VI), the peak of PHA-derived fluorescence in the histogram moved, as expected, from low FL1 intensities (gate G1) towards higher fluorescence intensities (gate G2). During this phase, the coefficient of variation (CV) of FL1, a value that gives information about the width of the peak and therefore about the spread of cellular PHA contents, resulted to be relatively small (<100%), suggesting a narrow distribution of intracellular PHA content across the population of cells (see supplementary materials Figure S1). In contrast, after the carbon source C8 was depleted (data points VII to X), a separation into two distinct populations took place. One cell cluster remained in the high FL1 intensity gate (G2), indicating a high PHA content, and a second cluster formed in the lower FL1 intensity gate (G1), suggesting the formation of a distinct population of cells with low or no intracellular PHA (Figure 3). Fluorescence scatter plots and several values for cell counts, fluorescence intensities, as well as the CV values for the overall measurements and for the gates G1 and G2 are shown in supplementary materials Figure S1.

**Figure 4:**
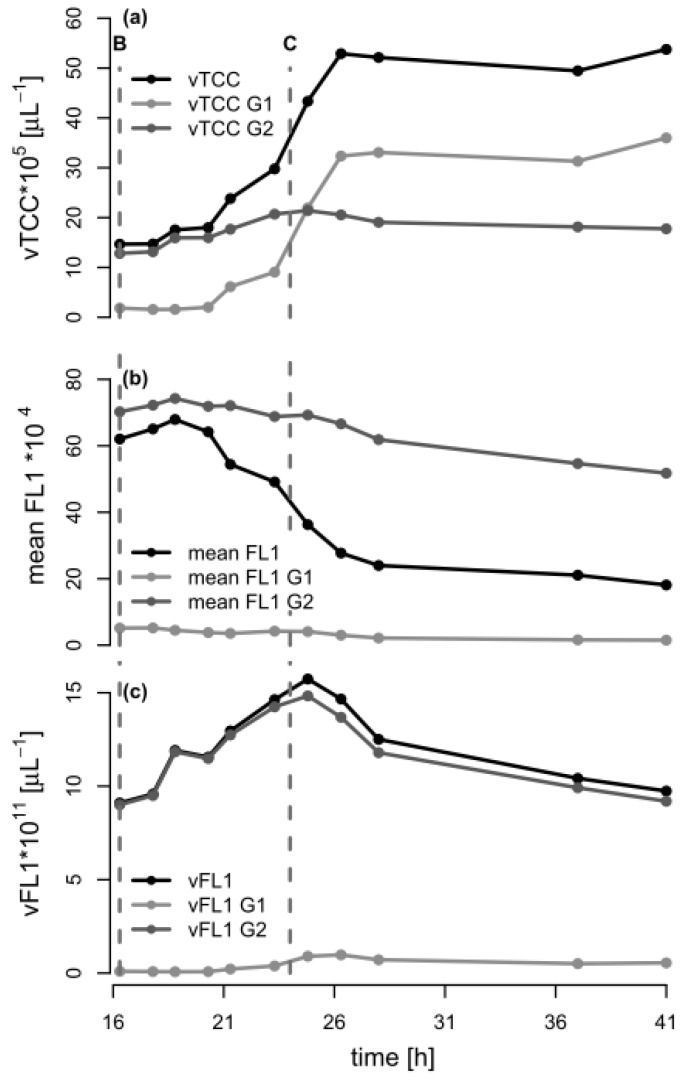
Analysis of the flow cytometry (FCM) data during the depolymerization phase of the fed-batch experiment with *P. putida* KT2440 on octanoic acid (C8). The data from the FCM analysis during the second phase of fermentation (*t* = 16.3–41 h) are shown for the total population, as well as for the populations of gates G1 and G2 (low/no PHA and high PHA content, respectively). The dashed lines B and C indicate the time points when the C8 feeding was stopped and the ammonium pulse added (16.3 h), and when C8 was completely consumed (*t* = 24 h), respectively. (**a**) Volumetric total cell count (vTCC); (**b**) Mean FL1 fluorescence (PHA content); (**c**) Volumetric fluorescence (vFL1, computed by multiplying vTCC by FL1).

In order to further analyze the mechanism behind the formation of subpopulations, we re-plotted the data of Figure 1 in terms of the volumetric total cell count (vTCC), FCM-determined average PHA content per cell (mean FL1, which would be the signal most similar to the PHA content determined by GC), and total volumetric PHA content (vFL1, the product of vTCC and mean FL1) with a focus on the depolymerization phase (Figure 4). Next, we repeated the data analysis, but used the cell numbers and the fluorescence values of the two subpopulations in the G1 and G2 gates (see Figure 3, supplementary materials Figure S1). This should allow for an understanding of the development of the total cell number and mean PHA in the total population in terms of the development of the two contributing subpopulations. Interestingly, the number of cells in G2 (high PHA content) showed only a small increase between 16.3 h (end of carbon feed) and 24 h (carbon source consumed), and then remained essentially constant. In contrast, the number of cells in G1 (low PHA content) increased more than 10-fold, until the total number of cells in G1 was about twice the number of cells in G2, about 2.3 h after the carbon source in the supernatant had been consumed (*t* = 26.3 h). This clearly suggests that the overall increase of the vTCC after 16.3 h (Figure 1a and Figure 4a) is mostly due to the formation of a subpopulation containing cells with a low PHA content. Furthermore, the PHA content (mean FL1) of the cells belonging to the high-PHA gate G2 remained constant between 16.3 h and 21 h, and then decreased by approximately 30% until the end of the experiment (Figure 4b). As a result, the volumetric PHA content increased proportionally with the number of cells in the high-PHA gate G2 until 24 h, and then decreased with the decreasing PHA content per cell, again in the G2 gate. However, it is striking that even though the PHA seems to have been mobilized to some extent in the G2 population, it was only the G1 population that increased in terms of vTCC, whereas the vTCC of the high-PHA content cells remained constant (Figure 4). These results strongly suggest that the apparent decrease in average PHA content between 20 h and 28 h, as shown in Figure 1c, is nearly entirely due to the formation of a low-PHA or even PHA-free subpopulation under conditions of carbon limitation.

**Figure 5:**
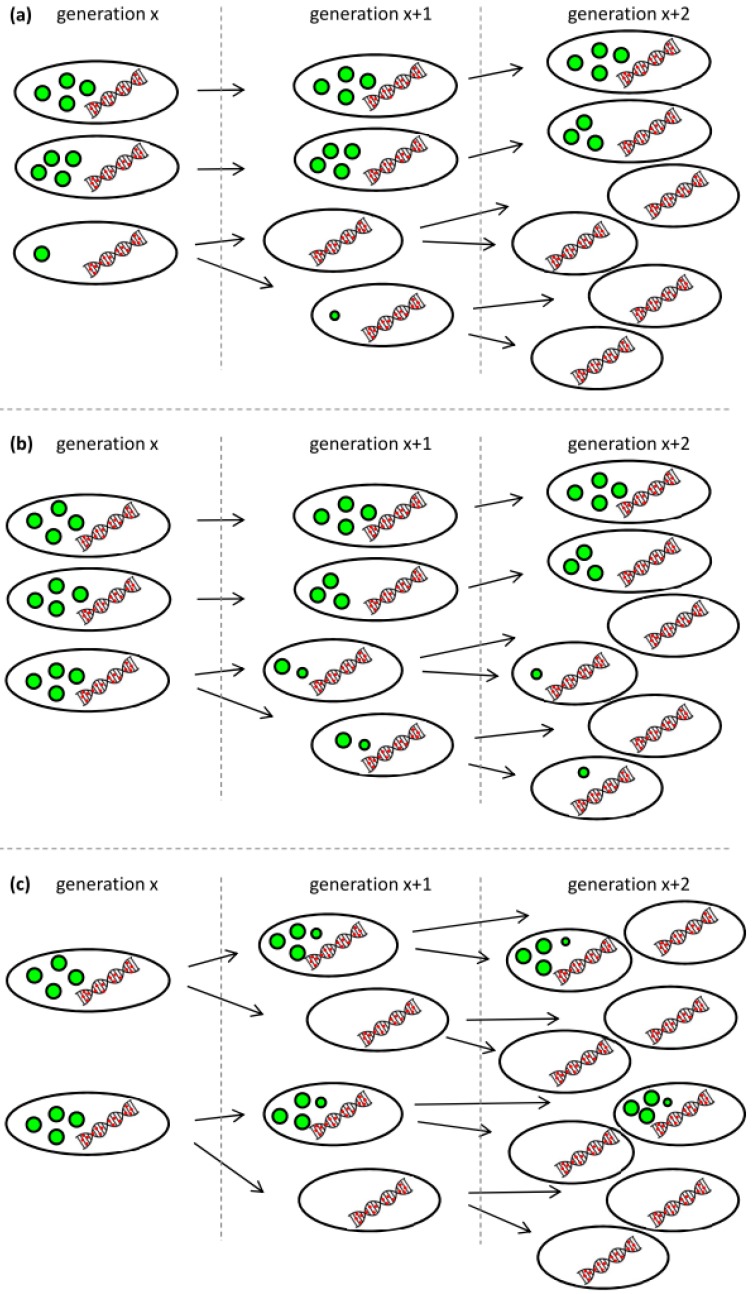
Possible scenarios leading to the occurrence of a low-PHA subpopulation at the onset of carbon limitation. (**a**) Low-PHA or PHA-free cells that are present in small concentrations divide faster than the cells that accumulated a lot of PHA and consume fatty acid monomers secreted from the PHA containing subpopulation; (**b**) Not all cells have the same propensity to recycle their PHA for further growth; (**c**) PHA granules are distributed asymmetrically when cells are dividing under conditions of carbon limitation. The colors were chosen according to the fluorescent staining for the analysis by flow cytometery: green represents the BODIPY 493/503-stained PHA granules, and red represents SYTO 62-stained nucleic acids. The amount and the size of the green granules symbolize the PHA content per cell.

We see three possible explanations for the observed behavior that are also illustrated in Figure 5. First, low-PHA cells that were present at very small concentrations already during the polymerization phase might have preferentially continued to divide at a higher growth rate when the lack of nitrogen did no longer inhibit cell division. The carbon source needed for the growth of this G1 population would have come from the small amount of the intracellular PHA of these cells, and from recycled 3-hydroxyalkanoic acid monomers secreted by cells of the G2 population as soon as the remaining carbon source in the supernatant was consumed (*t* = 24 h) (Figure 5a). Second, the ability to degrade intracellular PHA rapidly might have been distributed in an uneven manner among the PHA-containing population. This would mean that some cells would have degraded PHA in order to divide, whereas others would not have done so (Figure 5b). Indeed, in the critical phase when the carbon source starts to be limiting (Figure 3, sample VII at 23.3 h), we clearly see a transition peak of cells with an intermediate PHA content that is supposedly the basis of the G1 population that shows an increasing vTCC during this phase of the process. However, this transition population was only present during a very short period of time. Finally, PHA-containing cells might distribute the granules in which PHA is stored asymmetrically among the daughter cells when dividing (Figure 5c). This would lead in the extreme to one daughter cell with PHA and the other one without any or with only little PHA that would then appear in the G1 gate of the fluorescence histogram or scatterplot (Figure 3, supplementary materials Figure S1).

In this context, it is interesting to note that the heterogeneous distribution of PHA during cell division has been observed before, specifically in exponentially growing *phaF* deletion mutants of *P. putida* KT2442 [25,30], and in *phaM* deletion mutant *R. eutropha* cells [31,32]. PhaF and PhaM are proteins that are involved in the distribution of PHA granules into daughter cells during a cell division. However, in the case presented here, it is very unlikely that an asymmetric cell division occurred due to a mutation in PhaF, since cells were grown for only approximately 10 generations, and because of the reproducibility of the phenotype. It seems, therefore, much more plausible that the different growth condition, namely carbon limitation, favors a bistable growth and PHA distribution pattern according to one or several of the hypotheses mentioned above. Several studies mentioned above assessed the distribution of PHA granules under PHA accumulating conditions under carbon excess [25,26,30,31,32].

In general, PHA depolymerization is undesirable in industrial PHA production, since it causes a fast loss of valuable product. Hence, it is crucial to harvest the cells as close as possible to the time point when the PHA content is maximal. This time point can be determined by a sudden increase of the pO_2_ signal in aerobic processes, or in general with the here applied FCM-based analysis method. According to the data presented above, a first indication of PHA depolymerization is not necessarily detectable as a decrease in mean FL1, as would be expected, but rather by the formation of a low PHA subpopulation. In fact, it is clear that the vTCC in the G1 subpopulation starts to increase after 23.3 h, whereas only a minor change in the mean FL1 can be observed at this time (Figure 4). Interestingly, 23.3 h corresponds to the time point a few minutes prior to complete depletion of the carbon substrate. Therefore, a process operator can decide based on a fast FCM-based analysis (total analysis time in the order of 5 min) if the production process should be terminated, or, during the process, if the feeding rate needs to be adjusted. In comparison, the determination of the substrate concentration in the culture supernatant by HPLC takes at least 30 min (see Section 2.3.2), during which valuable PHA could already be lost by depolymerization.

## 4. Conclusions

Single cell analysis by flow cytometry of *P. putida* K2440 cells revealed that the nutritional state significantly influenced the distribution of PHA granules among the population. Expectedly, when cells were grown under carbon excess, PHA was accumulated and fluorescence measurements of stained, intracellular PHA showed a very homogeneous population in terms of PHA contents. However, bistability became apparent when the culture was subsequently exposed to carbon limitation and the population clearly separated into two main clusters. One cluster remained with a high amount of PHA, whereas a second cluster of cells with no or very little PHA was rising in number. This suggests either that PHA-free cells were able to grow faster by consuming fatty acid monomers released from PHA-degrading cells or that the cells were dividing in an asymmetric way into one daughter cell without PHA and the other one keeping all PHA of the mother cell.

## Supplementary Materials

The following are available online at http://www.mdpi.com/2306-5354/4/2/58/s1. Figure S1: Fluorescence scatter plots and histograms from flow cytometry measurements during the PHA polymerization-depolymerization experiment.
