# Supplementary material for: The Bistable Behaviour of Pseudomonas putida KT2440 during PHA Depolymerization under Carbon Limitation
Source: Bioengineering (Basel). 2017 Jun 19;4(2):58. doi: 10.3390/bioengineering4020058 (PMC5590454; doi:10.3390/bioengineering4020058)
Supplement: Supplementary file 1 [file bioengineering-04-00058-s001.pdf]

Supplmentary materials to the article: “The Bistable Behaviour of *Pseudomonas putida* KT2440 during PHA Depolymerization under Carbon Limitation” by S. Karmann et al.

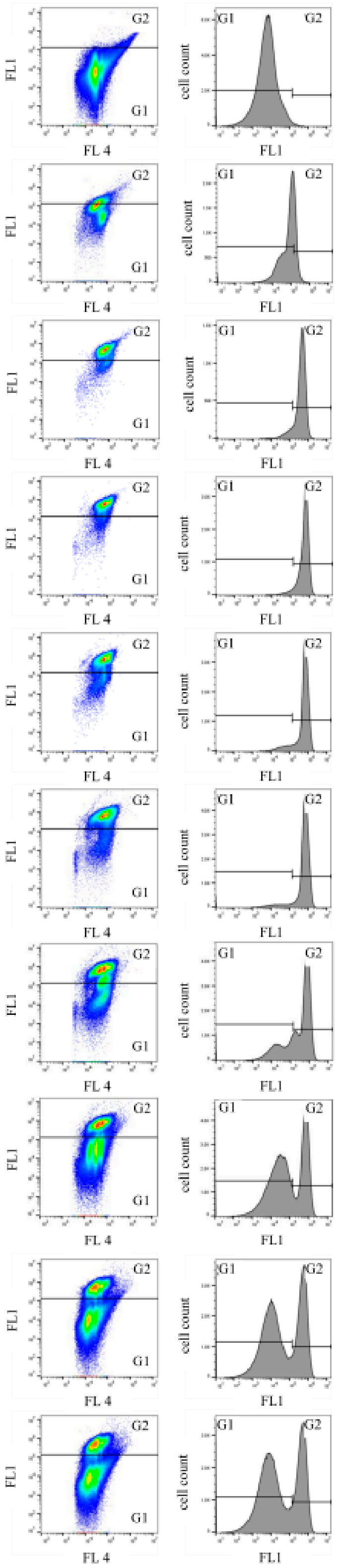

**I) 0 h**	total	G1	G2
vTCC [µL^{-1}]	$7.19*10^{4}$	$7.13*10^{3}$	$5.7*10^{2}$
% of plot	-	99.2	0.84
mean FL1	$1.28*10^{4}$	$9.50*10^{3}$	-
CV FL1 [%]	845	164	-

**II) 5 h**	total	G1	G2
vTCC [µL^{-1}]	$1.19*10^{5}$	$8.58*10^{4}$	$3.31*10^{4}$
% of plot	-	76.2	23.8
mean FL1	$1.02*10^{5}$	$6.81*10^{4}$	$2.04*10^{5}$
CV FL1 [%]	161	-	-

**III) 11 h**	total	G1	G2
vTCC [µL^{-1}]	$6.24*10^{5}$	$7.80*10^{4}$	$5.46*10^{5}$
% of plot	-	12.0	88.0
mean FL1	$4.29*10^{5}$	$6.25*10^{4}$	$4.80*10^{5}$
CV FL1 [%]	96	-	87

**IV) 14 h**	total	G1	G2
vTCC [µL^{-1}]	$1.27*10^{6}$	$9.40*10^{4}$	$1.18*10^{6}$
% of plot	-	7.1	92.9
mean FL1	$5.56*10^{5}$	$6.30*10^{4}$	$5.94*10^{5}$
CV FL1 [%]	57	-	50

**V) 16.3 h**	total	G1	G2
vTCC [µL^{-1}]	$1.47*10^{6}$	$1.85*10^{5}$	$1.28*10^{6}$
% of plot	-	12.4	87.6
mean FL1	$6.20*10^{5}$	$499*10^{4}$	$6.61*10^{5}$
CV FL1 [%]	59	-	45

**VI) 20.3 h**	total	G1	G2
vTCC [µL^{-1}]	$1.8*10^{6}$	$2.03*10^{5}$	$1.6*10^{6}$
% of plot	-	11.2	88.8
mean FL1	$6.42*10^{5}$	$3.42*10^{4}$	$7.25*10^{5}$
CV FL1 [%]	65	108	52

**VII) 23.3 h**	total	G1	G2
vTCC [µL^{-1}]	$2.98*10^{6}$	$9.06*10^{5}$	$2.07*10^{6}$
% of plot	-	30.0	70.0
mean FL1	$4.91*10^{5}$	$4.22*10^{4}$	$6.88*10^{5}$
CV FL1 [%]	95	97	62

**VIII) 24.8 h**	total	G1	G2
vTCC [µL^{-1}]	$4.34*10^{6}$	$2.19*10^{6}$	$2.14*10^{6}$
% of plot	-	51.6	49.4
mean FL1	$2.19*10^{5}$	$4.1*10^{4}$	$6.93*10^{5}$
CV FL1 [%]	124	92.0	65

**IX) 26.3 h**	total	G1	G2
vTCC [µL^{-1}]	$5.29*10^{6}$	$3.23*10^{6}$	$2.06*10^{6}$
% of plot	-	60.8	39.2
mean FL1	$2.77*10^{5}$	$2.96*10^{4}$	$6.62*10^{5}$
CV FL1 [%]	142	105	59

**X) 37 h**	total	G1	G2
vTCC [µL^{-1}]	$4.95*10^{6}$	$3.13*10^{6}$	$1.81*10^{6}$
% of plot	-	63.7	36.3
mean FL1	$2.10*10^{5}$	$1.61*10^{4}$	$5.47*10^{5}$
CV FL1 [%]	155	150	60.3

Figure S1: Fluorescence scatter plots and histograms from flow cytometry measurements during the PHA polymerization - depoly-merization experiment. The panels show scatter plots of green BODIPY 493/503 fluorescence (FL1, PHA) and red SYTO 62 fluorescence (FL4, DNA) and their corresponding histograms for FL1. The samples I – X were taken throughout the bioprocess depicted in Figure 1. The gates G1 and G2, low and high FL1 values, respectively, were defined based on the scatter plot from the initial time point and help to visualize the evolution of the FL1 signal. The tables next to the panels comprise the respective values of volumetric total cell count (vTCC), mean FL1 and its coefficient of variation (CV) for the total population and the two gates.
